# Elevating local concentrations of GPIIb–IIIa antagonists counteracts platelet thrombus stability

**DOI:** 10.1007/s11239-012-0814-7

**Published:** 2012-10-17

**Authors:** Henry E. Speich, Ronit R. Furman, Lindsey T. Lands, Geoffrey D. Moodie, Lisa K. Jennings

**Affiliations:** 1 Department of Internal Medicine, Vascular Biology Center of Excellence, The University of Tennessee Health Science Center, 956 Court Avenue Coleman H300, Memphis, TN 38163 USA; 2 Department of Immunology, Microbiology and Biochemistry, The University of Tennessee Health Science Center, Memphis, TN 38163 USA; 3 Department of Surgery, The University of Tennessee Health Science Center, Memphis, TN 38163 USA; 4 Atrium Medical Corporation, Hudson, NH 03051 USA

**Keywords:** Abciximab, Arterial thrombosis, Eptifibatide, GPIIb–IIIa antagonists, Intracoronary administration, Platelets

## Abstract

Glycoprotein IIb–IIIa (GPIIb–IIIa) antagonists have the capacity to destabilize coronary thrombi and restore vessel patency. Antagonist concentration and residence time, which can be increased by local intracoronary (LIC) administration, and thrombus age may be key factors that influence thrombus stability. Light transmission aggregometry was used to examine the effects of exposing human platelet aggregates to extremely high local levels of GPIIb–IIIa antagonists versus conventional therapeutic levels in vitro. Freshly-formed or aged platelet aggregates were subjected to GPIIb–IIIa antagonists (abciximab, eptifibatide) or direct thrombin inhibitor bivalirudin at concentrations simulating either conventional intravenous (IV) or LIC administration. The degree of antagonist-induced disaggregation was significantly higher using elevated (LIC) doses versus conventional (IV) doses (60.1 % vs. 7.4 % for abciximab, 41.6 % or 45.3 % vs. 17.6 % for eptifibatide, *p* < 0.01). Bivalirudin did not promote disaggregation. Microscopy confirmed noticeably smaller, more dispersed aggregates for antagonist LIC treatments. Dosing at LIC levels also induced more disaggregation than IV levels when aggregates were aged for 30 min prior to exposure. An in vitro perfusion model was used to simulate the fluid dynamics of IV or LIC administration of abciximab using a microporous local drug delivery balloon catheter such as the Atrium ClearWay™ RX. The perfusion model resulted in more rapid thrombus clearance with LIC dosing levels compared to IV. In summary, boosting the concentration of GPIIb–IIIa antagonists enhances dispersal of human platelet aggregates in vitro. These data provide a foundation for investigating increased local concentrations of GPIIb–IIIa antagonists in patients, as with LIC administration.

## Introduction

Platelets play a vital role in primary hemostasis. While circulating platelets are normally quiescent, they can be rapidly stimulated upon adherence to exposed subendothelial matrix components and by soluble agonists at sites of vascular injury. A complex interplay of adhesive interactions and intracellular signaling pathways, including granule secretion and cytoskeletal reorganization, facilitate platelet activation and aggregation to form a platelet-rich thrombus that arrests bleeding. Furthermore, platelet activation and aggregation contribute to the amplification of coagulation, clot retraction, and eventual wound healing processes [1]. The rupture of vulnerable plaque formed by atherosclerotic disease can lead to pathological thrombus formation and vascular occlusion, which may manifest dangerously as myocardial infarction in the coronary artery or occlusive stroke as a consequence of embolization.

Pharmacological intervention directed toward platelets has traditionally aimed to prevent platelet activation and subsequent thrombosis. Platelet glycoprotein IIb–IIIa (GPIIb–IIIa) receptor antagonists have proven to be potent anti-platelet agents with established clinical benefit to patients presenting with acute coronary events when either managed medically or undergoing percutaneous coronary intervention (PCI) [2, 3]. Numerous in vitro, ex vivo, and clinical studies clearly outline the capability of these agents to prevent platelet aggregation by inhibiting fibrinogen-mediated crosslinking of activated platelets via the activated GPIIb–IIIa receptor [4–6]. Recent studies reveal that these agents may have additional effects on platelets aggregates that have already formed, causing them to disengage and disperse [7–11]. Previous work in our laboratory confirmed that GPIIb–IIIa antagonists abciximab and eptifibatide readily dispersed freshly-formed platelet aggregates [12]. The mechanism for decreased aggregate stability correlated with antagonist-induced dissociation of fibrinogen from the platelet surface. The choice of antagonist, antagonist concentration, and age of the platelet aggregates affected the extent of platelet disaggregation.

The current study examined the capacity for extremely high local concentrations of abciximab and eptifibatide, as may be achieved through local intracoronary (LIC) administration, to disperse preformed platelet aggregates in vitro. The influence of aggregate stability on the effects of antagonist-induced disaggregation was also investigated by exposing aged platelet aggregates to the antagonists at LIC levels. Bivalirudin, a direct thrombin inhibitor, was included in these studies as a comparator because it is approved for use as an anticoagulant in patients with unstable angina undergoing percutaneous coronary angioplasty with or without provisional GPIIb–IIIa antagonist use. However, bivalirudin has not been reported to act directly on platelets to disrupt preformed thrombus. Lastly, an in vitro capillary perfusion model was employed to investigate the disaggregating effects of IV and LIC abciximab administration. This model was designed to simulate pertinent fluid dynamic conditions and respective local drug concentrations achieved by either standard IV administration or by LIC delivery through the ClearWay™ RX microporous drug delivery balloon catheter (Atrium Medical Corporation, Hudson NH). LIC delivery offers several potential advantages to traditional intracoronary (IC) delivery. Traditional IC delivery utilizes a guide catheter to infuse at the coronary ostium, limiting penetration into occlusive thrombus and allowing the drug to follow lower resistance pathways such as the aorta or the not-target coronary artery. In contrast, the ClearWay™ RX system atraumatically occludes proximal blood flow at the deployment site while delivering a therapeutic through a micropourous PTFE membrane. The agent is delivered at high concentration directly to a thrombus or as a distally advancing column of fluid between the device and the thrombus (when deployed proximally). Elevated drug levels are undiluted by upstream flow and maintained until the balloon is collapsed. Maximizing the effective drug concentration and dwell time at the site of delivery may enhance the potential for platelet disaggregation compared to the traditional method of intracoronary drug delivery.

## Materials and methods

### Materials

Unless otherwise specified, materials were obtained from Sigma-Aldrich (St. Louis, MO). d-Phenylalanyl-l-prolyl-l-arginine chloromethyl ketone (PPACK) anticoagulant was obtained from CalBiochem (La Jolla, CA). Eptifibatide (Merck, West Point, PA), 2 mg/mL solution, and abciximab (Eli Lilly, Indianapolis, IN), 2 mg/mL solution, were obtained in their commercially available packaging and formulations.

### Preparation of platelets suspensions

Blood was obtained by venipuncture from normal, consenting adult donors into PPACK anticoagulant and then centrifuged at 135 g for 15 min to obtain platelet-rich plasma (PRP). Remaining blood was centrifuged at 2,500*g* for 15 min to obtain platelet-poor plasma (PPP). Platelet concentration in PRP was measured with Z2 Particle Count and Size Analyzer (Beckman Coulter, Miami, FL). Platelet count was adjusted to 2.5 × 10^8^ platelets/mL with autologous PPP.

### Disaggregation of collagen-induced aggregates

Turbidometric light transmission aggregometry (LTA) was performed on a dual-channel lumiaggregometer (Payton Scientific, Buffalo, NY) to quantify the extent of collagen-induced platelet aggregation or disaggregation prior to and following exposure to GPIIb–IIIa antagonists or respective vehicle controls. A 500 μL aliquot of autologous PPP was used to blank each aggregometer. Test samples of PRP were aliquoted at 450 μL in aggregometer cuvettes. Aggregation was induced by addition of 50 μL of 20 μg/mL type I collagen (Chrono-Log, Havertown, PA), for a final concentration of 2 μg/mL. Aggregation was allowed to proceed for 3.5 min following agonist addition, a point which typically represented the maximum extent of aggregation. A novel technique was employed so that very high concentrations of antagonists in commercially available stock solutions or appropriate vehicle control dilutions could be used while maintaining physiological concentrations of platelets. For fresh aggregate experiments, stirring was halted after 3.5 min and aggregates were allowed to settle in the aggregometer cuvette for 1 min. Next, 400 μL of plasma was removed from the sample and discarded without disturbing the settled aggregates. The volume removed was replaced with 400 μL of autologous PPP, drug, and/or vehicle control. Stirring was then immediately resumed, and disaggregation response was recorded for 15 min. Identical methodology was employed in aged aggregate experiments, except that samples were allowed to settle and incubate at 37 °C for 30 min, instead of 1 min, before the 400 μL aliquot of plasma was removed and drug or control was introduced. For each experiment, the extent of light transmission through the sample at maximum aggregation was compared with the transmission at the resumption of stirring to confirm the stability of the formed aggregates.

Antagonist concentrations included in these studies represented those that are clinically relevant, approximating plasma levels following conventional intravenous administration of the drug in question. Concentrations of drug that might be achieved through intracoronary administration through a typical catheter system or through an intracoronary delivery system were also studied. The descriptive labels used in various results figures refer to the final concentration of the respective agent in the aggregometry cuvette. The concentration of 2 μg/mL abciximab was chosen to approximate the mean plasma level of abciximab immediately after a bolus IV administration [13]. Likewise, 2 μM eptifibatide and 11 μg/mL bivalirudin were chosen based on literature references to their respective mean plasma levels following IV administration [14–17]. The higher concentration of abciximab used was the highest concentration possible in this experimental system, obtained by replacing plasma removed from the aggregometry cuvette with an equal volume of full-strength stock abciximab. Due to stock eptifibatide low pH (~pH 5.3), the drug must be buffered prior to intracoronary administration. The 1 mM eptifibatide doses represent a 1:2.4 dilution of the stock eptifibatide in autologous PPP, relevant if operators choose to buffer the intracoronary bolus with autologous blood. The 1.6 mM eptifibatide dose was formulated by buffering the stock eptifibatide with sodium bicarbonate according to the method of Deibele et al. [18]. The 5 mg/mL bivalirudin concentration was based on a literature reference to traditional intracoronary administration of bivalirudin [19].

### Quantification of platelet disaggregation

Percent platelet aggregation (%PA) was determined 3.5 min after agonist addition (%PA_max_), at resumption of stirring immediately after antagonist addition to preformed aggregates (%PA_resume_), and at 5, 10, and 15 min after antagonist addition to preformed aggregates (%PA_time point_). The following calculations were made for %PA and percent platelet disaggregation (%PD):

*%PA* = *(distance on aggregometer tracing from 0 % [PRP] to maximum aggregation response at given time point)/(distance on tracing from 0 % [PRP] to 100 % aggregation [PPP])* *×* *100;*

$$ \% {\text PD} = (\% {\text{PA}}_{\text{resume}} -\% {\text{PA}}_{\text{time \; point}} )/(\% {\text{PA}}_{ \hbox{max} } ) $$

%PD was normalized by subtracting respective vehicle control %PD for each treatment.

### Analyses of aggregates by microscopy

The wells of a 24-well cell culture plate were preloaded with 55 μL of 10 % paraformaldehyde, pH 7.4. After disaggregation extent was recorded for 15 min in LTA experiments, the entire 500 μL sample was removed from the aggregometer cuvette and placed into a plate well. The sample volume was then gently pipetted three times to achieve 1 % final paraformaldehyde concentration. Control aliquots of untreated PRP diluted 10 % with saline, and aggregates at maximum aggregation (3.5 min post-agonist addition) were also prepared. Photographs of PRP or aggregates were taken at 40× magnification within 36 h of fixation.

### Capillary perfusion aggregometry

A capillary perfusion system, detailed previously, was used to simulate exposure of freshly-formed, platelet-rich thrombi to either steady-state IV levels of GPIIb–IIIa antagonists or to a LIC bolus infusion of antagonist followed by subsequent steady-state exposure under relevant fluid dynamic conditions [12, 20]. Briefly, whole blood anticoagulated with PPACK was incubated with rhodamine 6G to fluorescently label platelets and leukocytes. Labeled blood was drawn from a reservoir via syringe pump through a rectangular capillary coated with human type III collagen at a flow rate corresponding to normal arterial shear stress of 40 dynes/cm^2^ [21, 22]. Plasma von Willebrand Factor (vWF) and platelets in the perfused blood encountered and adhered to the collagen-coated surface, stimulating formation of platelet-rich thrombus. The extent of thrombus formation was tracked by quantifying the pixel intensity across a series of images recorded with a fluorescent videomicroscope focused on the capillary.

Following thrombus formation, precisely as the initial volume of blood in the reservoir was depleted, blood pretreated with carrier control or antagonist or a bolus of stock antagonist was added to the reservoir, thus simulating an infusion of drug without disruption of flow. For simulations of IV drug administration, blood pretreated with abciximab at 2 μg/mL or with vehicle control was used and flow was continued at normal arterial shear stress. For simulations of LIC administration, a bolus of abciximab was introduced at 2 mg/mL and flow adjusted for 1 min to the unique fluid dynamic conditions associated with intracoronary drug administration via the ClearWay™ RX therapeutic infusion catheter. This bolus was followed by a steady-state phase with normal arterial fluid dynamics at IV abciximab levels to simulate rapid systemic dispersal of the bolus.

The vehicle or drug treatments and the fluid dynamic parameters used during the infusion and steady state portions of the perfusion experiments are summarized in Table 1. The wall shear stress required to replicate intracoronary delivery was calculated using Hagen-Poiseuille methodology and based on theoretical ClearWay™ RX infusion at a flow rate 10 mL/min in a 3 mm diameter vessel in vivo. Fluid dynamic assumptions included that flow was laminar, noncompressible, and Newtonian, and that the viscosity of the bolus infusion was approximately that of water (~1cP) rather than that of whole blood (~3.6cP) [22, 23]. These assumptions account for the delivery of drugs in aqueous vehicle with upstream blood flow completely occluded. The wall shear stress target for the infusion was determined to be 0.7 dynes/cm^2^ and was achieved by adjusting the capillary perfusion rate based on calculations for flow in a rectangular channel [23].

**Table 1:** Drug concentrations and shear stress values during bolus and steady state segments of perfusion experiments

	Treatment	Bolus concentration	Bolus shear stress (dynes/cm^2^)	Steady state concentration	Steady state shear stress (dynes/cm^2^)
1	IV carrier control	0.1 % PBS in blood	40	0.1 % PBS	40
2	IV abciximab	2 μg/mL in blood	40	2 μg/mL	40
3	IC abciximab	2 mg/mL in PBS	0.7	2 μg/mL	40

### Statistical analysis

Data were analyzed using Microsoft Excel and SigmaStat (Version 3.5, Systat Software Inc., San Jose, CA) software packages. All values are reported as mean ± standard deviation. Analysis of variance (ANOVA) and Student–Newman Keuls or Holm-Sidak multiple comparison procedures were used to determine statistical differences between groups, with *p* < 0.05 considered statistically significant.

## Results

**Fig. 1:**
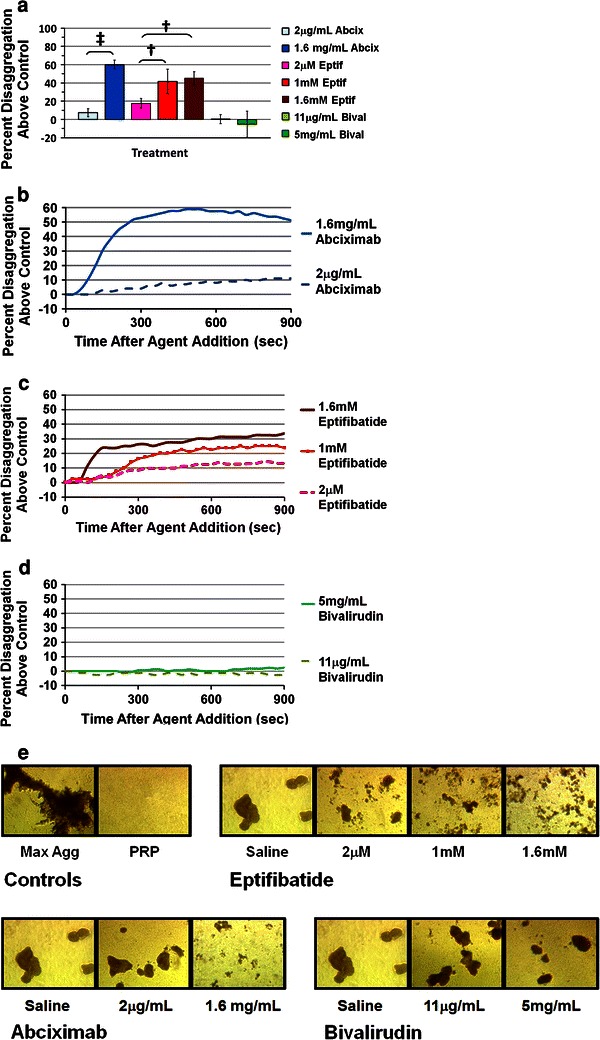
Platelet disaggregation following exposure to GPIIb–IIIa antagonists (abciximab or eptifibatide) or bivalirudin. **a** Percent disaggregation (normalized to respective vehicle control) at 15 min post-drug addition. Results are expressed as mean ± SD (*error bars*), *n* = 4, ^**‡**^ *p* < 0.001, ^**†**^ *p* < 0.01 compared to lowest experimental dose of the respective drug. **b–d** Normalized disaggregation* curves* from a single representative donor for each drug. **e** Bright field microscopy images of platelet aggregates fixed with paraformaldehyde at 15 min post-drug addition (40× magnification). Images are from a single donor that was representative of an *n* = 4

Collagen is one of the initial agonists encountered by platelets during vascular injury; therefore, platelet aggregation was initiated by exposing a stirring suspension of platelets to 2 μg/mL type I collagen and monitoring the formation and stability of platelet aggregates using LTA. The mean aggregation response for normal adult donors was 78.6 ± 1.6 % (*n* = 4). Once maximal aggregation was achieved at 3.5 min, the freshly-formed aggregates were exposed to pre-specified concentrations of GPIIb–IIIa antagonists or respective vehicle controls and the extent of disaggregation was measured at 5, 10 and 15 min after addition of the antagonist. The results for abciximab, eptifibatide, and bivalirudin treatments are depicted in Fig. 1. Treatment with the LIC equivalent concentration of 1.6 mg/mL abciximab resulted in a significantly higher extent of platelet disaggregation above respective vehicle control when compared to IV concentration of 2 μg/mL abciximab (60.1 vs. 7.4 %, *p* < 0.001) at 15 min (Fig. 1a). Likewise, exposure of aggregates to either 1 mM eptifibatide or to 1.6 mM eptifibatide resulted in significantly more disaggregation when compared to exposure to the IV concentration of 2 μM eptifibatide at 15 min [41.6 % (1.0 mM) or 45.3 % (1.6 mM) vs. 17.6 %, *p* < 0.01]. In contrast, the extent of disaggregation using a therapeutic concentration of 11 μg/mL bivalirudin was not significantly different from treatment with IC 5 mg/mL bivalirudin (Fig. 1a). In fact, neither bivalirudin concentration was effective at inducing significant amounts of disaggregation (data not shown, *p* > 0.05).

Platelet disaggregation curves for representative donors plotted over the course of an entire experiment illustrate that disaggregation proceeded at more rapid rates for the highest concentrations of abciximab and eptifibatide compared to the lowest doses of each respective agent (Fig. 1b, c). Bivalirudin treatment, at either concentration, consistently resulted in little or no disaggregation above control (Fig. 1d). Representative images of platelet aggregates fixed at the 15 min time point and visualized with brightfield microscopy confirmed that treatment with the elevated concentrations of antagonists lead to greater aggregate dispersal when compared to the lower doses (Fig. 1e). Conversely, bivalirudin treated aggregates were not visually different than vehicle treated aggregates. Representative images are also shown for aggregates fixed at 3.5 min following initiation of aggregation and for unstimulated platelets.

**Fig. 2:**
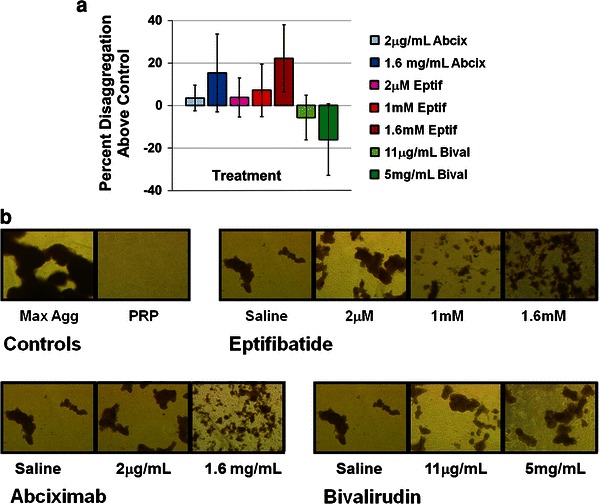
Disaggregation of aged platelet aggregates following exposure to GPIIb–IIIa antagonists (abciximab or eptifibatide) or bivalirudin. **a** Percent disaggregation (normalized to vehicle control) at 15 min post-drug addition. Results are expressed as mean ± SD (*error bars*), *n* = 4. **b** Bright field microscopy images of platelet aggregates fixed with paraformaldehyde at 15 min post-drug addition (40× magnification). Images are from a single donor that was representative of an *n* = 4

The next series of aggregometry experiments utilized collagen-induced platelet aggregates that had been aged for 30 min prior to introduction of the abciximab, eptifibatide, bivalirudin, or respective vehicle controls. The persistence of stable aged aggregates after 30 min of rest, prior to antagonist exposure, has been previously documented for this technique, and was visually confirmed for all samples in this study [12, 20]. Exposure of aged aggregates to the greatly increased doses of abciximab and eptifibatide consistently resulted in greater extents of disaggregation above vehicle than did treatment with the low doses of the antagonists (Fig. 2a). The overall extents of antagonist-induced platelet disaggregation were lower than that observed with fresh aggregates after 30 min of aging. Representative bright field microscopy images qualitatively agree with the LTA data, showing more disperse aggregates at high antagonist concentrations compared to treatment with low concentrations of antagonist or vehicle control (Fig. 2b). Bivalirudin treatment did not induce platelet disaggregation above that observed for respective vehicle controls.

**Fig. 3:**
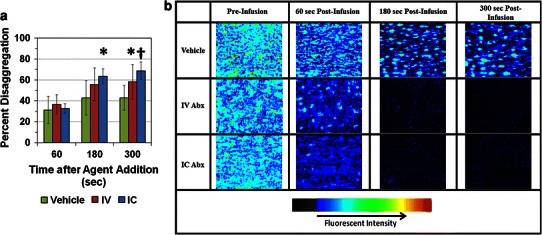
Capillary perfusion assay for platelet disaggregation following exposure to GPIIb–IIIa antagonist abciximab. **a** Percent disaggregation after saline control bolus and infusion (vehicle), 2 μg/mL abciximab bolus followed by 2 μg/mL abciximab infusion (IV), or 2 mg/mL abciximab bolus with IC equivalent fluid dynamics followed by 2 μg/mL abciximab infusion (IC). **b** False color images of rhodamine-labeled platelet thrombi before treatment infusion and at 1, 3, and 5 min post-infusion of control or drug. **a** and **b** results are expressed as mean ± SD (*error bars*). *p < 0.05, ^**†**^ *p* < 0.01 compared to vehicle. For all panels, *n* = 5 for vehicle, *n* = 6 for IV abciximab, and *n* = 7 for IC abciximab

The disaggregating potential of abciximab was further investigated in a capillary perfusion system. Eptifibatide was omitted due to buffering considerations and bivalirudin was not considered due to lack of disaggregating activity in LTA experiments. Adherent, platelet-rich thrombi were exposed to fluid dynamic conditions and drug concentrations simulating either conventional IV administration of the drug or LIC administration via the ClearWay™ RX microporous drug delivery balloon catheter. Exposure of the preformed thrombi to both abciximab treatment simulations resulted in significant thrombus reduction at the 5 min time point following abciximab exposure compared to the vehicle control for no antagonist exposure (Fig. 3a). However, the simulation of LIC abciximab treatment led to significant disaggregation compared control at 3 min of exposure, whereas the extent of disaggregation for the IV administration simulation was not significant at that time, indicating a more rapid onset of action for the LIC treatment. False-color images of thrombi for representative experimental runs also suggest that abciximab treatment induces platelet dispersal, with an earlier onset of action for the LIC treatment regime (Fig. 3b).

## Discussion

Recent investigations by our laboratory and others suggest that GPIIb–IIIa antagonists possess the ability to disengage pre-formed platelet aggregates [8–12, 24]. Antagonist-induced dissociation of fibrinogen from the platelet surface implied that these agents regulate the interaction of the GPIIb–IIIa receptor with fibrinogen even after the initial stages of fibrinogen binding [12]. Antagonist-mediated dispersal might also be affected by the underlying aggregate stability prior to antagonist exposure. Platelet aggregates freshly-formed in response to 5 μM ADP were more readily dispersed by abciximab or eptifibatide than aggregates formed by the more potent stimulation of 2 μg/mL collagen. Furthermore, higher concentrations of both antagonists than typically achieved following conventional IV administration were necessary to affect collagen-induced aggregates that had been aged for 30 min prior to antagonist exposure [12]. These interesting observations provided an impetus to further pursue the questions of whether GPIIb–IIIa antagonist-induced platelet aggregate instability may contribute to the observed clinical effectiveness of these agents, and if this newly-revealed mode of action can be exploited as an intervention for pre-existing thrombus burden.

GPIIb–IIIa binds to fibrinogen exclusively via the fibrinogen γ chain, despite the fact that multiple RGD motifs are present on fibrinogen α chains. GPIIb–IIIa can bind other RGD-containing ligands, such as vWF, at a site that has been proposed to be nonidentical and spatially distinct from the fibrinogen binding locale [25]. Hu et al. utilized a surface plasmon residence technique to evaluate models of competitive inhibition between the two proposed ligand binding sites of GPIIb–IIIa [26]. This work suggested a two-site model of allosteric competitive inhibition between fibrinogen and RGD-type ligands. Importantly though, while RGD-type ligand binding influenced the association and dissociation of fibrinogen, fibrinogen binding did not affect the association or dissociation of RGD-type ligand. RGD ligands were able to bind to the receptor even when fibrinogen was already bound and induced the dissociation of fibrinogen. These observations provide a rationale for the dissociation of platelet-bound fibrinogen and platelet disaggregation observed following exposure to tirofiban or lamifiban, both nonpeptide RGD mimetics [10, 11, 27]. Structural and binding studies later conclusively determined that the binding site of the fibrinogen γC peptide to GPIIb–IIIa overlaps the RGD binding site and suggested that eptifibatide, developed to mimic the KGD sequence from the γC peptide, binds similarly to the γC peptide and RGD, but enters the GPIIb–IIIa binding pocket from a different angle [28, 29]. Eptifibatide-induced dissociation of fibrinogen and platelet disaggregation might therefore proceed by a mechanism similar to that observed for RGD-mimetics [10–12]. Abciximab is a chimeric monoclonal antibody fragment that does not directly compete with fibrinogen for binding to GPIIb–IIIa, but is instead thought to operate through steric hindrance or allosteric mechanisms [30, 31]. Abciximab-induced platelet disaggregation has been observed in vitro by several investigators [8, 10, 11]. Although abciximab promotes fibrinogen dissociation from the platelet surface, the molecular basis for the dissociation is unclear at this time [12].

In addition to the differences in the mechanisms of receptor antagonism by abciximab and eptifibatide, other antagonist-specific structural and pharmacological properties may influence the ability of each agent to promote platelet disaggregation. The molecular mass of abciximab is approximately 50 times larger than the mass of eptifibatide which could reduce diffusion and transport within aggregates and thrombi. Abciximab also has a very high affinity and low dissociation constant, with a half-life of dissociation from the receptor of up to 4 h. Conversely, eptifibatide is a lower affinity competitive antagonist and rapidly dissociates from the receptor within minutes [31]. Pharmacological differences between antagonists presumably led to greater extents of platelet disaggregation by eptifibatide compared to abciximab when levels were near IV therapeutic concentrations [12]. However, in the current study, both abciximab and eptifibatide rapidly and substantially dissociated platelet aggregates when administered at elevated levels simulating intracoronary administration.

The temporal dynamics of the GPIIb–IIIa/fibrinogen interaction, as well as the dynamic changes occurring over time in the thrombus are likely to have dramatic effects on the efficacy of pharmacologically-based thrombus dispersal in vivo. Parise and Phillips noted that the reversibility of ^125^I-fibrinogen binding to reconstituted GPIIb–IIIa receptors decreased after 1 h [32]. Marguerie et al. also described a two-phase interaction between fibrinogen and intact platelets with an initial reversible phase followed by irreversible binding within 30 min [33]. Advances in intravital microscopy and other techniques have revealed complex spatial and temporal changes in platelet morphology and activation during thrombus formation in flowing blood [34–37]. An activated core of platelets consolidates and stabilizes over time at the site of vessel injury while unactivated discoid platelets form the bulk of the initial thrombus. The decrease in the reversibility of receptor/fibrinogen interaction and the decreased likelihood of disrupting a fully consolidated platelet-rich thrombus could explain why early administration of eptifibatide has been associated with improved TIMI 3 patency before primary PCI as compared with late (just before PCI) or no administration [38]. Similar studies also demonstrate that early administration of abciximab or tirofiban versus late administration of those agents results in improved outcomes in patients with ST Segment Elevation Myocardial Infarction (STEMI) [39–42]. These insights led us to explore the influence of thrombus aging on the effectiveness of antagonist induced aggregate dispersal. Our data demonstrate that aggregate dispersal by levels of antagonists corresponding to local concentrations following LIC delivery is more pronounced than that observed for IV levels even after aggregates have been allowed to stabilize. Therefore, LIC administration of antagonist may be more effective therapeutically when treating patients presenting after the onset of acute coronary symptoms related to an occlusive event.

Recent clinical studies emphasize that novel strategies that improve reperfusion by reducing thrombus burden and or microembolization may result in improved clinical outcomes. The Thrombus Aspiration during PCI in Acute myocardial infarction Study (TAPAS) found that enhanced reperfusion after thrombus aspiration, assessed by myocardial blush grade or resolution of ST-segment elevation at 30 days and at 1 year, significantly lowered rates of death and major cardiac events compared to conventional treatments [43, 44]. The current study supports the mechanistic rationale for positive clinical outcomes in studies assessing the value of reperfusion strategies utilizing intracoronary administration of GPIIb–IIIa antagonists. A meta-analysis by Hansen et al. concluded that traditional IC administration can improve outcomes compared to IV administration in acute coronary syndromes (ACS) undergoing PCI, especially patients with STEMI undergoing primary PCI [45]. A meta-analysis by Friedland et al. included 11 randomized clinical trials comparing traditional IC and IV administration of abciximab, eptifibatide, or tirofiban in ACS. The analysis concluded that IC administration of GPIIb–IIIa antagonists has favorable effects on TIMI flow, target vessel revascularization, and short-term mortality after PCI, with no difference in rates of bleeding [46]. The AIDA STEMI trial, with 2,065 randomized patients, is the largest clinical trial conducted thus far comparing intracoronary versus intravenous abciximab administration during PCI in patients with acute STEMI [47]. While the study confirmed the safety of intracoronary abciximab administration, no differences in the combined endpoint of death, reinfarction, or congestive heart failure were observed between IV and IC administrations. However, intracoronary drug delivery was done proximally through a guide catheter in that study, allowing portions of the bolus to be diverted to the aorta and non-target arteries. Drug delivered by this technique is also rapidly diluted and transported downstream by blood flowing past the guide catheter, reducing penetration into the targeted thrombus. The AIDA STEMI authors’ discussion and independent commentary cite several other possible explanations for the lack of benefit reported in that study, including the study being underpowered due to a lower than expected incidence of the primary endpoint and differences in design versus previous smaller-scale studies [47, 48].

The perfusion assay described in this study was included because coronary fluid dynamics ultimately regulates the convective and diffusive transport of both endogenous mediators of thrombosis and exogenous therapeutic agents to, from, and within a site of thrombosis, as well as mediate the shear forces that naturally work against thrombus accumulation. For this assay we chose to simulate a catheter-based intracoronary delivery system designed to maximize local drug concentration and dwell time at the site of delivery, parameters which our earlier study revealed were important mediators of antagonist-induced thrombus dissolution [12]. The ClearWay™ RX microporous drug delivery balloon catheter occludes the target vessel upon infusion at the site of thrombosis prior to local drug delivery through the microporous balloon. Fluid dynamic and drug concentration calculations in the current simulation assumed downstream blood flow was completely occluded during drug administration, i.e. local drug concentration is not diluted by blood flowing past the expanded balloon at the treatment site and drug dwell time are maximized throughout the procedure. This study used in vitro models to examine the effects of IIb/IIIa inhibitors on platelet aggregation, and as such certain aspects present in vivo (e.g. interactions with endothelial cells) may not be captured in this model.

Prati et al. report that local delivery of abciximab through the ClearWay™ RX system reduces thrombus burden in ACS patients and that reperfusion, as measured by corrected TIMI frame count, and clinical outcomes were improved compared with abciximab delivered to the coronary through a guide catheter [49]. Deibele and Gibson detail two case reports concerning successful intracoronary application of this technology and describe increased TIMI grade flow and dissolution in response to intracoronary administration of eptifibatide [50]. The recently completed INFUSE-AMI study compared the effect IC abciximab versus no abciximab on infarct size in in high-risk patients with STEMI presenting early [51]. Design measures were incorporated to isolate the effects of bolus-only abciximab, including eliminating the 12-h post-PCI abciximab infusions that were present in both IC and IV AIDA STEMI groups and using procedural anticoagulation with bivalirudin without routine glycoprotein IIb/IIIa inhibition in the control group. Additionally, the ClearWay™ Rx catheter was used to deliver abciximab directly to the infarct lesion site. INFUSE-AMI concluded that intracoronary abciximab significantly reduced the primary study endpoint, infarct size, at 30 days in patients with anterior STEMI reperfused early, while other indices of myocardial reperfusion, including ST-segment resolution and myocardial blush grade, were unaffected. Another multicenter randomized study to evaluate LIC abciximab administration with the ClearWay™ RX catheter to improve outcomes with lysis, the IC ClearLy study, is underway [52].

We observed no significant dispersal of fresh or aged platelet aggregates in response to low or elevated concentrations of the direct thrombin inhibitor bivalirudin. This result was not surprising, given that bivalirudin has not been reported to influence the interaction between platelets and pre-bound ligand. The ACUITY and ISAR-REACT-2 trial data support the utilization of GPIIb–IIIa antagonists in troponin-positive patients, particularly if upstream clopidogrel is not administered [53, 54]. In ACUITY, patients randomized to bivalirudin alone consistently tended to have substantially higher ischemic event rates than did GPIIb–IIIa antagonist treated patients if clopidogrel was initiated more than 30 min after PCI completion or not at all [55]. These results are presumably due to bivalirudin acting as an anticoagulant only, without a direct, platelet-related mechanism that targets thrombus formation and stability.

In summary, platelet-mediated thrombus formation is a complex and dynamic phenomenon. Recent research has shed light on the unique contributions of blood rheology to platelet-mediated thrombus formation, as well as brought new emphasis on the spatial and temporal parameters that affect arterial thrombus stability [36, 37]. The interplay between the GPIIb–IIIa receptor, fibrinogen, and GPIIb–IIIa antagonists, as well as the time-course of platelet aggregate stabilization is critical for understanding how pharmacological perturbation of preformed platelet aggregates might be optimized. The current study provides in vitro evidence for greater aggregate dispersal by LIC equivalent concentrations of GPIIb–IIIa antagonist which supports and helps explain why clinical studies have reported improved reperfusion and patient outcomes when these agents are administered via the intracoronary route rather than intravenously.

## References

[1] Jennings LK (2009). Role of platelets in atherothrombosis. Am J Cardiol.

[2] Kong DF, Califf RM, Miller DP, Moliterno DJ, White HD, Harrington RA, Tcheng JE, Lincoff AM, Hasselblad V, Topol EJ (1998). Clinical outcomes of therapeutic agents that block the platelet glycoprotein IIb/IIIa integrin in ischemic heart disease. Circulation.

[3] Ortolani P, Marzocchi A, Marrozzini C, Palmerini T, Saia F, Taglieri N, Baldazzi F, Dall’Ara G, Nardini P, Gianstefani S, Guastaroba P, Grilli R, Branzi A (2009). Long-term effectiveness of early administration of glycoprotein IIb/IIIa agents to real-world patients undergoing primary percutaneous interventions: results of a registry study in an ST-elevation myocardial infarction network. Eur Heart J.

[4] Topol EJ, Byzova TV, Plow EF (1999). Platelet GPIIb–IIIa blockers. Lancet.

[5] Lincoff AM, Califf RM, Topol EJ (2000). Platelet glycoprotein IIb/IIIa receptor blockade in coronary artery disease. J Am Coll Cardiol.

[6] Coller BS, Shattil SJ (2008). The GPIIb/IIIa (integrin alphaIIbbeta3) odyssey: a technology-driven saga of a receptor with twists, turns, and even a bend. Blood.

[7] Collet JP, Montalescot G, Lesty C, Soria J, Mishal Z, Thomas D, Soria C (2001). Disaggregation of in vitro preformed platelet-rich clots by abciximab increases fibrin exposure and promotes fibrinolysis. Arterioscler Thromb Vasc Biol.

[8] Marciniak SJ, Mascelli MA, Furman MI, Michelson AD, Jakubowski JA, Jordan RE, Marchese PJ, Frelinger AL (2002). An additional mechanism of action of abciximab: dispersal of newly formed platelet aggregates. Thromb Haemost.

[9] Collet JP, Montalescot G, Lesty C, Weisel JW (2002). A structural and dynamic investigation of the facilitating effect of glycoprotein IIb/IIIa inhibitors in dissolving platelet-rich clots. Circ Res.

[10] Moser M, Bertram U, Peter K, Bode C, Ruef J (2003). Abciximab, eptifibatide, and tirofiban exhibit dose-dependent potencies to dissolve platelet aggregates. J Cardiovasc Pharmacol.

[11] Goto S, Tamura N, Ishida H (2004). Ability of anti-glycoprotein IIb/IIIa agents to dissolve platelet thrombi formed on a collagen surface under blood flow conditions. J Am Coll Cardiol.

[12] Speich HE, Earhart AD, Hill SN, Cholera S, Kueter TJ, Smith JN, White MM, Jennings LK (2009). Variability of platelet aggregate dispersal with glycoprotein IIb–IIIa antagonists eptifibatide and abciximab. J Thromb Haemost.

[13] Quinn MJ, Murphy RT, Dooley M, Foley JB, Fitzgerald DJ (2001). Occupancy of the internal and external pools of glycoprotein IIb/IIIa following abciximab bolus and infusion. J Pharmacol Exp Ther.

[14] Robson R, White H, Aylward P, Frampton C (2002). Bivalirudin pharmacokinetics and pharmacodynamics: effect of renal function, dose, and gender. Clin Pharmacol Ther.

[15] Gilchrist IC (2003). Platelet glycoprotein IIb/IIIa inhibitors in percutaneous coronary intervention: focus on the pharmacokinetic-pharmacodynamic relationships of eptifibatide. Clin Pharmacokinet.

[16] Gretler DD, Guerciolini R, Williams PJ (2004). Pharmacokinetic and pharmacodynamic properties of eptifibatide in subjects with normal or impaired renal function. Clin Ther.

[17] Koster A, Spiess B, Jurmann M, Dyke CM, Smedira NG, Aronson S, Lincoff MA (2006). Bivalirudin provides rapid, effective, and reliable anticoagulation during off-pump coronary revascularization: results of the “EVOLUTION OFF” trial. Anesth Analg.

[18] Deibele AJ, Kirtane AJ, Pinto DS, Lucca MJ, Neva C, Shui A, Murphy SA, Tcheng JE, Gibson CM (2006). Intracoronary bolus administration of eptifibatide during percutaneous coronary stenting for non ST elevation myocardial infarction and unstable angina. J Thromb Thrombolysis.

[19] Cortese B, Picchi A, Micheli A, Limbruno U (2009). Intracoronary bivalirudin for no reflow reversal: a second chance to treat this disorder?. J Thromb Thrombolysis.

[20] Speich HE, Grgurevich S, Kueter TJ, Earhart AD, Slack SM, Jennings LK (2008). Platelets undergo phosphorylation of Syk at Y525/526 and Y352 in response to pathophysiological shear stress. Am J Physiol Cell Physiol.

[21] Slack SM, Turitto VT (1993). Chapter 2 Fluid dynamic and hemorheologic considerations. Cardiovasc Pathol.

[22] Slack SM, Turitto VT (1994). Flow chambers and their standardization for use in studies of thrombosis. On behalf of the subcommittee on rheology of the scientific and standardization committee of the ISTH. Thromb Haemost.

[23] Slack SM, Turitto VT (1993). Fluid dynamic and hemorheologic considerations. Cardiovasc Pathol.

[24] Collet JP, Montalescot G, Lesty C, Mishal Z, Soria J, Choussat R, Drobinski G, Soria C, Pinton P, Barragan P, Thomas D (2001). Effects of Abciximab on the architecture of platelet-rich clots in patients with acute myocardial infarction undergoing primary coronary intervention. Circulation.

[25] Bennett JS (2001). Platelet-fibrinogen interactions. Ann N Y Acad Sci.

[26] Hu DD, White CA, Panzer-Knodle S, Page JD, Nicholson N, Smith JW (1999). A new model of dual interacting ligand binding sites on integrin alphaIIbbeta3. J Biol Chem.

[27] Frojmovic M, Labarthe B, Legrand C (2005). Inhibition and reversal of platelet aggregation by alphaIIbbeta3 antagonists depends on the anticoagulant and flow conditions: differential effects of Abciximab and Lamifiban. Br J Haematol.

[28] Springer TA, Zhu J, Xiao T (2008). Structural basis for distinctive recognition of fibrinogen gammaC peptide by the platelet integrin alphaIIbbeta3. J Cell Biol.

[29] Hantgan RR, Stahle MC, Lord ST (2010). Dynamic regulation of fibrinogen: integrin alphaIIbbeta3 binding. Biochemistry.

[30] Artoni A, Li J, Mitchell B, Ruan J, Takagi J, Springer TA, French DL, Coller BS (2004). Integrin beta3 regions controlling binding of murine mAb 7E3: implications for the mechanism of integrin alphaIIbbeta3 activation. Proc Natl Acad Sci USA.

[31] Schror K, Weber AA (2003). Comparative pharmacology of GP IIb/IIIa antagonists. J Thromb Thrombolysis.

[32] Jirouskova M, Jaiswal JK, Coller BS (2007). Ligand density dramatically affects integrin alpha IIb beta 3-mediated platelet signaling and spreading. Blood.

[33] Marguerie GA, Edgington TS, Plow EF (1980). Interaction of fibrinogen with its platelet receptor as part of a multistep reaction in ADP-induced platelet aggregation. J Biol Chem.

[34] Maxwell MJ, Westein E, Nesbitt WS, Giuliano S, Dopheide SM, Jackson SP (2007). Identification of a 2-stage platelet aggregation process mediating shear-dependent thrombus formation. Blood.

[35] Nesbitt WS, Westein E, Tovar-Lopez FJ, Tolouei E, Mitchell A, Fu J, Carberry J, Fouras A, Jackson SP (2009). A shear gradient-dependent platelet aggregation mechanism drives thrombus formation. Nat Med.

[36] Jackson SP, Nesbitt WS, Westein E (2009). Dynamics of platelet thrombus formation. J Thromb Haemost.

[37] Munnix IC, Cosemans JM, Auger JM, Heemskerk JW (2009). Platelet response heterogeneity in thrombus formation. Thromb Haemost.

[38] Zeymer U, Zahn R, Schiele R, Jansen W, Girth E, Gitt A, Seidl K, Schroder R, Schneider S, Senges J (2005). Early eptifibatide improves TIMI 3 patency before primary percutaneous coronary intervention for acute ST elevation myocardial infarction: results of the randomized integrilin in acute myocardial infarction (INTAMI) pilot trial. Eur Heart J.

[39] Cutlip DE, Ricciardi MJ, Ling FS, Carrozza JP, Dua V, Garringer J, Giri S, Caputo RP (2003). Effect of tirofiban before primary angioplasty on initial coronary flow and early ST-segment resolution in patients with acute myocardial infarction. Am J Cardiol.

[40] Gyongyosi M, Domanovits H, Benzer W, Haugk M, Heinisch B, Sodeck G, Hodl R, Gaul G, Bonner G, Wojta J, Laggner A, Glogar D, Huber K (2004). Use of abciximab prior to primary angioplasty in STEMI results in early recanalization of the infarct-related artery and improved myocardial tissue reperfusion—results of the Austrian multi-centre randomized ReoPro-BRIDGING Study. Eur Heart J.

[41] van ‘t Hof AW, Ernst N, de Boer MJ, de Winter R, Boersma E, Bunt T, Petronio S, Marcel Gosselink AT, Jap W, Hollak F, Hoorntje JC, Suryapranata H, Dambrink JH, Zijlstra F (2004). Facilitation of primary coronary angioplasty by early start of a glycoprotein 2b/3a inhibitor: results of the ongoing tirofiban in myocardial infarction evaluation (On-TIME) trial. Eur Heart J.

[42] Godicke J, Flather M, Noc M, Gyongyosi M, Arntz HR, Grip L, Gabriel HM, Huber K, Nugara F, Schroder J, Svensson L, Wang D, Zorman S, Montalescot G (2005). Early versus periprocedural administration of abciximab for primary angioplasty: a pooled analysis of 6 studies. Am Heart J.

[43] Svilaas T, Vlaar PJ, van der Horst IC, Diercks GF, de Smet BJ, van den Heuvel AF, Anthonio RL, Jessurun GA, Tan ES, Suurmeijer AJ, Zijlstra F (2008). Thrombus aspiration during primary percutaneous coronary intervention. N Engl J Med.

[44] Vlaar PJ, Svilaas T, van der Horst IC, Diercks GF, Fokkema ML, de Smet BJ, van den Heuvel AF, Anthonio RL, Jessurun GA, Tan ES, Suurmeijer AJ, Zijlstra F (2008). Cardiac death and reinfarction after 1 year in the Thrombus Aspiration during Percutaneous coronary intervention in Acute myocardial infarction Study (TAPAS): a 1-year follow-up study. Lancet.

[45] Hansen PR, Iversen A, Abdulla J (2010). Improved clinical outcomes with intracoronary compared to intravenous abciximab in patients with acute coronary syndromes undergoing percutaneous coronary intervention: a systematic review and meta-analysis. J Invasive Cardiol.

[46] Friedland S, Eisenberg MJ, Shimony A (2011). Meta-analysis of randomized controlled trials of intracoronary versus intravenous administration of glycoprotein IIb/IIIa inhibitors during percutaneous coronary intervention for acute coronary syndrome. Am J Cardiol.

[47] Thiele H, Wohrle J, Hambrecht R, Rittger H, Birkemeyer R, Lauer B, Neuhaus P, Brosteanu O, Sick P, Wiemer M, Kerber S, Kleinertz K, Eitel I, Desch S, Schuler G (2012). Intracoronary versus intravenous bolus abciximab during primary percutaneous coronary intervention in patients with acute ST-elevation myocardial infarction: a randomised trial. Lancet.

[48] Bertrand OF, Jolly S (2012). AIDA STEMI: no benefit for intracoronary abciximab. Lancet.

[49] Prati F, Capodanno D, Pawlowski T, Ramazzotti V, Albertucci M, La Manna A, Di Salvo M, Gil RJ, Tamburino C (2010). Local delivery versus intracoronary infusion of abciximab in patients with acute coronary syndromes. JACC Cardiovasc Interv.

[50] Deibele AJ, Michael Gibson C (2011). Intracoronary delivery of eptifibatide with the ClearWay(R) RX infusion catheter. Catheter Cardiovasc Interv.

[51] Stone GW, Maehara A, Witzenbichler B, Godlewski J, Parise H, Dambrink JH, Ochala A, Carlton TW, Cristea E, Wolff SD, Brener SJ, Chowdhary S, El-Omar M, Neunteufl T, Metzger DC, Karwoski T, Dizon JM, Mehran R, Gibson CM (2012). Intracoronary abciximab and aspiration thrombectomy in patients with large anterior myocardial infarction: the INFUSE-AMI randomized trial. JAMA.

[52] Sardella G, Sangiorgi GM, Mancone M, Colantonio R, Donahue M, Politi L, Ducci CB, Carbone I, Francone M, Ligabue G, Fiocchi F, Di Roma A, Benedetti G, Lucisano L, Stio RE, Agati L, Modena MG, Genuini I, Fedele F, Gibson M (2010). A multicenter randomized study to evaluate intracoronary abciximab with the ClearWay catheter to improve outcomes with Lysis (IC ClearLy): trial study design and rationale. J Cardiovasc Med (Hagerstown).

[53] Stone GW, McLaurin BT, Cox DA, Bertrand ME, Lincoff AM, Moses JW, White HD, Pocock SJ, Ware JH, Feit F, Colombo A, Aylward PE, Cequier AR, Darius H, Desmet W, Ebrahimi R, Hamon M, Rasmussen LH, Rupprecht HJ, Hoekstra J, Mehran R, Ohman EM (2006). Bivalirudin for patients with acute coronary syndromes. N Engl J Med.

[54] Kastrati A, Mehilli J, Neumann FJ, Dotzer F, ten Berg J, Bollwein H, Graf I, Ibrahim M, Pache J, Seyfarth M, Schuhlen H, Dirschinger J, Berger PB, Schomig A (2006). Abciximab in patients with acute coronary syndromes undergoing percutaneous coronary intervention after clopidogrel pretreatment: the ISAR-REACT 2 randomized trial. JAMA.

[55] Lincoff AM, Steinhubl SR, Manoukian SV, Chew D, Pollack CV, Feit F, Ware JH, Bertrand ME, Ohman EM, Desmet W, Cox DA, Mehran R, Stone GW (2008). Influence of timing of clopidogrel treatment on the efficacy and safety of bivalirudin in patients with non-ST-segment elevation acute coronary syndromes undergoing percutaneous coronary intervention: an analysis of the ACUITY (Acute Catheterization and Urgent Intervention Triage strategY) trial. JACC Cardiovasc Interv.

